# Prognostic significance of TOP2A in non-small cell lung cancer revealed by bioinformatic analysis

**DOI:** 10.1186/s12935-019-0956-1

**Published:** 2019-09-11

**Authors:** Wenxia Ma, Bin Wang, Yaping Zhang, Ziyue Wang, Dan Niu, Siyu Chen, Zhirong Zhang, Ningning Shen, Weixia Han, Xiaoqin Zhang, Rong Wei, Chen Wang

**Affiliations:** Department of Pathology, The Second Hospital of ShanXi Medical University, No. 382 WuYi Road, Tai Yuan, 030000 Shanxi China

**Keywords:** Non-small cell lung cancer (NSCLC), TOP2A, GEO database, Different expressed genes (DEGs), Protein–protein interaction network (PPI), Survival analysis, Biomarker

## Abstract

**Background:**

Lung cancer has been a common malignant tumor with a leading cause of morbidity and mortality, current molecular targets are woefully lacking comparing to the highly progressive cancer. The study is designed to identify new prognostic predictors and potential gene targets based on bioinformatic analysis of Gene Expression Omnibus (GEO) database.

**Methods:**

Four cDNA expression profiles GSE19188, GSE101929, GSE18842 and GSE33532 were chosen from GEO database to analyze the differently expressed genes (DEGs) between non-small cell lung cancer (NSCLC) and normal lung tissues. After the DEGs functions were analyzed, the protein–protein interaction network (PPI) of DEGs were constructed, and the core gene in the network which has high connectivity degree with other genes was identified. We analyzed the association of the gene with the development of NSCLC as well as its prognosis. Lastly we explored the conceivable signaling mechanism of the gene regulation during the development of NSCLC.

**Results:**

A total of 92 up regulated and 214 down regulated DEGs were shared in four cDNA expression profiles. Based on their PPI network, TOP2A was connected with most of other genes and was selected for further analysis. Kaplan–Meier overall survival analysis (OS) revealed that TOP2A was associated with worse NSCLC patients survival. And both GEPIA analysis and immunohistochemistry experiment (IHC) confirmed that TOP2A was aberrant gain of expression in cancer comparing to normal tissues. The clinical significance of TOP2A and probable signaling pathways it involved in were further explored, and a positive correlation between TOP2A and TPX2 expression was found in lung cancer tissues.

**Conclusion:**

Using bioinformatic analysis, we revealed that TOP2A could be adopted as a prognostic indicator of NSCLC and it potentially regulate cancer development through co-work with TPX2. However, more detailed experiments are needed to clarify its drug target role in clinical medical use.

## Background

Lung cancer has been a malignant tumor with a leading cause of both morbidity and mortality worldwide [1–3], and 80–85% is non-small cell lung cancer (NSCLC), with different biological processes and pathological appearance to the other 10–15% small cell lung cancer (SCLC) [4]. NSCLC includes lung adenocarcinoma, squamous cell carcinoma and large cell carcinoma. Although cancer is still a challenging and incurable disease, the up-rising new therapies including immunotherapy and targeted therapies are bringing promising effect to the clinical patients treatment [5]. Especially in lung adenocarcinoma, great improvement is taking place in targeted therapies, nearly ten genes have been developed as drug targets, including epidermal growth factor receptor (EGFR), anaplasticlymphoma kinase (ALK), ROS1, RET, HER2, BRAF, PIK3CA, Kras, Nras and MET [6–8] the drugs that are developed based on these genes expression situation are all showing exciting curative effect [8–14].

However, the available ten bio-targets are still numbered as opposed to the highly heterogeneous, complicated and progressive cancer development [4, 15–17]. As a well known fact that the main reason responsible for the incurability of cancer is their fast “adaptive” to outer environment changes, malignant tumors posses ever-changing characteristics according to different clinical treatments [9, 18]. Not to mention the other subtypes of NSCLC besides adenocarcinoma, including squamous carcinoma and large cell carcinoma, the drug targets are woefully numbered currently. For instance, in the squamous carcinoma, only FGFR2 and DDR2 are known to be aberrantly mutated and could be developed into potential clinical use as drug targets, but as for now, both drugs are still in clinical trial stage [19]. As for the large cell carcinoma, there is none probable drug target yet [20]. It is of vital importance to keep identifying new prognostic biomarkers and other potential gene targets [21].

Recently, great advance is happening to high-throughput technologies, bringing in tremendous amount of clinical data, which provides a rich source for researchers to better understand the molecular basis of cancer development and to identify disease-causing gene alterations thus exploring potential drug targets for therapeutic intervention [22–24]. Large portion of these data are public open and accessible to world wide researchers. Bioinformatic is a data-driven branch of science, with many of the algorithms and databases developed to analyze different types of data [25]. A lot of analysis tools including software, databases and website services are powerful and free [25–28], although some software are commercial, they can be purchased at a virtually very low cost by school students and education institutes teachers [29].

In the study, multiple bioinformatic tools were applied to analyze the four cDNA expression profiles from Gene Expression Omnibus (GEO) database including GSE19188, GSE101929, GSE18842 and GSE33532. Firstly, GEO2R tool was used to detect the differently expressed genes (DEGs) between NSCLC and normal lung tissues, the DEGs that were shared in all four profiles were chosen. Secondly, the protein–protein interaction (PPI) network of shared DEGs was constructed using Cytoscape3.6.0 software, and the core gene with highest connectivity degree with other genes was identified. Then, the correlation with NSCLC patients overall survival rate (OS) was evaluated with KM plotter online databases and clinical significance was analyzed based on immunohistochemistry experiment (IHC) results data. Last but not least, the potential function signaling behind the core gene’s regulation on NSCLC development was preliminary explored and genes that co-work with it were explored using STRING, Oncomine and GEPIA. The results shall provide delightful insights to the unearth of prognostic biomarker candidates and new potential bio targets to NSCLC patients.

## Materials and methods

### Data source: cDNA expression profiles from GEO database

Four cDNA expression profiles GSE19188 [30], GSE101929 [31], GSE18842 [32] and GSE33532 [33] were chosen from GEO online public database [34] based on the sample size and their publication time (we mainly focused on the profiles that contains at least 20 paired samples and those being publicated recently). And GSE19188 profile was based on GPL570[HG-U133_Plus_2] Affymetrix Human Genome U133 Plus 2.0 Array, containing 91 NSCLC samples and 20 normal lung tissues. GSE101929 was based on GPL570[HG-U133_Plus_2] Affymetrix Human Genome U133 Plus 2.0 Array, containing 32 NSCLC samples and 34 normal lung samples. GSE18842 was based on GPL570[HG-U133_Plus_2] Affymetrix Human Genome U133 Plus 2.0 Array, containing 46 NSCLC samples and 45 normal lung samples. And GSE33532 was based on GPL570[HG-U133_Plus_2] Affymetrix Human Genome U133 Plus 2.0 Array, containing 80 NSCLC samples and 20 normal lung samples.

### Identification of DEGs between NSCLC and normal lung tissue

To analyze the DEGs between NSCLC and normal lung tissues, GEO2R tool [35], which is a public interactive online service was used in four cDNA profiles respectively. The criteria for DEGs selection were set as adjusted P value < 0.05 and |log2FC| ≥ 2. And E Chart online service for Venn diagram was then used to screen the DEGs that were shared in all four cDNA profiles. Meanwhile, GO and KEGG were used to preliminary analyze the main biological processes, molecular functions and signaling pathways the DEGs enriched in.

### PPI network construction and core gene identification

To construct the PPI network of shared DEGs, the Search Tool for the Retrieval of Interacting Genes (STRING) was used, which is an online database for searching the direct (physical) and indirect (functional) association between various proteins. STRING contains the information of 9643763 proteins from 2031 species up to now [27, 36]. The cut-off criteria to construct the network was set as confidence score ≥ 0.4 and maximum number of interactors = 0. Additionally, the top gene with highest connectivity degree with surrounding genes was picked based on the PPI network by Cytoscape3.6.0 software [37].

### Kaplan–Meier survival analysis

Kaplan–Meier plotter is an open access online service for the overall survival analysis of various cancers including lung cancer, breast cancer, gastric and ovarian cancer, as well as hepatocellular carcinoma, containing a total of 10,461 patients samples and their clinical information [38, 39]. In the study, we used Kaplan–Meier plotter to analyze the correlation between TOP2A gene and NSCLC patients OS, followed by drawing the survival curve. Additionally, the clinical and mRNA transcription data including 574 lung adenocarcinoma and 555 lung squamous cell carcinoma were downloaded from TCGA database for multivariate COX regression analysis and exploring TOP2A expression relationship with clinical parameters.

### GEPIA analysis of gene expression

GEPIA is a newly developed online software, which is based on the sequencing database of 9736 cancer and 8587 normal samples from TCGA and GTEx programs. The software is commonly used for analyzing certain genes expression differences between cancer and normal tissues in various tumor types [40, 41]. In the study, we used GEPIA to preliminary explore the expression differences of TOP2A between NSCLC and normal lung samples.

### Immunohistochemistry (IHC) experiment regents and tissue samples

All of the clinical patients sample were stored in our biobank, and they were all collected from routine surgeries at General Surgery Department and sent for pathology examination at the Department of Pathology of local Hospital. Informed consent from the patients as well as approval by the Hospital Institutional Board were obtained (ShanXi, China).

IHC experiment was performed on VENTANA platform (Roche), the TOP2A recombinant primary rabbit monoclonal antibody (SY27-00) was purchased from Invitrogen, secondary antibody (Envision/HRP kit) and DAB detection kit were from ZSBG-Bio. Other reagents including H_2_O_2_, phosphate-buffered saline (PBS) and hematoxylin stain were from the hospital supply department.

### Immunohistochemistry (IHC) experiment protocol

IHC experiment was conducted to confirm the gene expression between NSCLC and normal lung tissues using 107 cases of biobank cancer samples following the experimental procedure as below.

The 107 paraffin-embedded tissue were made in tissue arrays first and made to slices. The stored slices were firstly taken out of refrigerator and rewarmed at room temperature for 20 min, followed by the deparaffin, rehydration and a 10 min boiling in 10 mmol/l citrate buffer for antigen retrieval. The sections would then be soaked in methanol containing 0.3% H_2_O_2_ for 10 min with the purpose of inhibiting of endogenous peroxidase activity. After being blocked with bovine serum albumin in PBS for 30 min, the sections would be incubated with primary antibody (dilution 1:250) for 2 h at 37 °C in Biochemistry Cultivation Cabinet, and another 40 min at 37 °C with species-specific secondary antibodies labeled with horseradish peroxidase (HRP) and finally visualized in DAB followed by the counterstaining of nuclei with hematoxylin.

### Evaluation of IHC results

The relative TOP2A protein expression level was evaluated according to both the tissue section’s staining intensity and staining area. The intensity and area of immunostaining was scored by two experienced pathologists in our department with no prior knowledge of the clinical and pathological details of the patients. Nuclear staining was regarded as positive according to TOP2A antibody specification sheet. The staining intensity was classified based on the following criteria: none (0), mild (1), moderate (2) and strong (3). And the staining area was stratified as follows: < 5% (0), 6–25% (1), 26–50% (2), 51–75% (3) and > 75% (4). The final TOP2A expression level in each sample was scored by multiply the staining intensity by staining area, using the score = 6 as cutoff point, final score < 6 was defined as negative, and score ≥ 6 was classified as positive [42].

Additionally, the gene’s clinical significance was analyzed based on the clinical data of above 107 patients.

### Related signaling pathways and co-work genes mining

The Oncomine database is a web-based data mining platform that incorporates 264 independent datasets for collecting, standardizing, analyzing, and delivering transcriptomic cancer data for biomedical research [43]. In the study, we used Oncomine for analyzing the various expression levels of TOP2A in different subtypes of NSCLC and exploring the co-expression genes relating to TOP2A. As for the TOP2A expression in subtypes of NSCLC, the query terms were set as: ① analysis type: lung cancer vs normal analysis; ② GENE: TOP2A; and for the co-expression analysis, the query terms were set as: ① GENE: TOP2A; ② analysis type: co-expression analysis; ③ non small cell lung cancer.

### RNA extraction and quantity real-time PCR (qRT-PCR)

Total mRNA of 30 lung adenocarcinoma and 30 lung squamous cell carcinoma samples were extracted using RNAiso-Plus (TAKARA, DaLian, China), and mRNA of matched adjacent normal tissue of each cancer sample were also extracted. cDNA was then synthesized from 1 μg extracted mRNA using cDNA synthesis kit (TAKARA, DaLian, China) according to the manufacturer’s instructions. Real-time PCR was performed on Roche z 480 with primers as:

TOP2A:

Former: CATTGAAGACGCTTCGTTATGG

Reverse: CAGAAGAGAGGGCCAGTTGTG

TPX2:

Former: CTTCCAATCACCGTCCCC

Reverse: TATTTCCACAGTTCTTGCCTCT

GAPDH:

Former: AGAAGGCTGGGGCTCATTTG

Reverse: AGGGGCCATCCACAGTCTTC

The cycling conditions were: 95 °C 5 min for 1 cycle; 95 °C 5 s, 60 °C 30 s, and 72 °C 34 s for 40 cycles followed by the melting curve stage. The relative expression of TOP2A and TPX2 were evaluated based on the 2^−ΔΔCT^ calculation, each sample get three replicates.

### Statistical analysis

Chi-square test was used to analyze the relationship between TOP2A expression and NSCLC clinicopathological features. T-test was used to analyze the relative mRNA expression of TOP2A and TPX2 in qPT-PCR experiment, and Pearson analysis was performed for exploring the connection between TOP2A and TPX2 genes. P < 0.05 was considered statistically significant.

## Results

### Identification of 306 DEGs shared by four GEO profiles

**Fig. 1:**
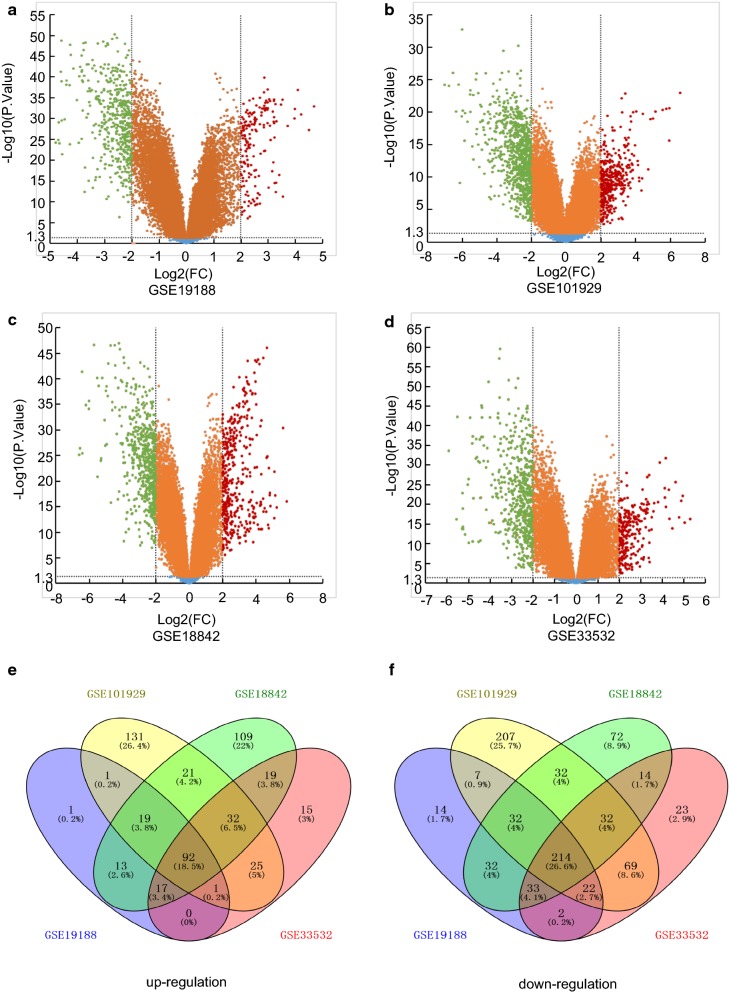
The DEGs screened from four GEO expression profiles. Up-regulated (red-colored spots) and down-regulated (green-colored spots) DEGS in NSCLC comparing to normal lung tissues were screened from GEO profiles **a** GSE19188, **b** GSE101929, **c** GSE18842 and **d** GSE33532 respectively. **e** 92 up-regulated and **f** 214 down-regulated DEGs were shared by four GEO expression profiles

We chose four cDNA expression profiles GSE18842, GSE19188, GSE33532 and GSE101929 from GEO database to analyze the DEGs between NSCLC and normal lung tissues. And a total of 1029, 635, 795 and 1304 DEGs including 419, 170, 248, 428 up-regulated and 610, 465, 547, 876 down-regulated genes were identified in GSE18842, GSE19188, GSE33532 and GSE101929 respectively (Fig. 1a–d). Meanwhile, a whole of 306 DEGs including 92 up-regulated and 214 down-regulated genes were shared in all four profiles shown by the Venn diagram (Fig. 1e, f).

**Fig. 2:**
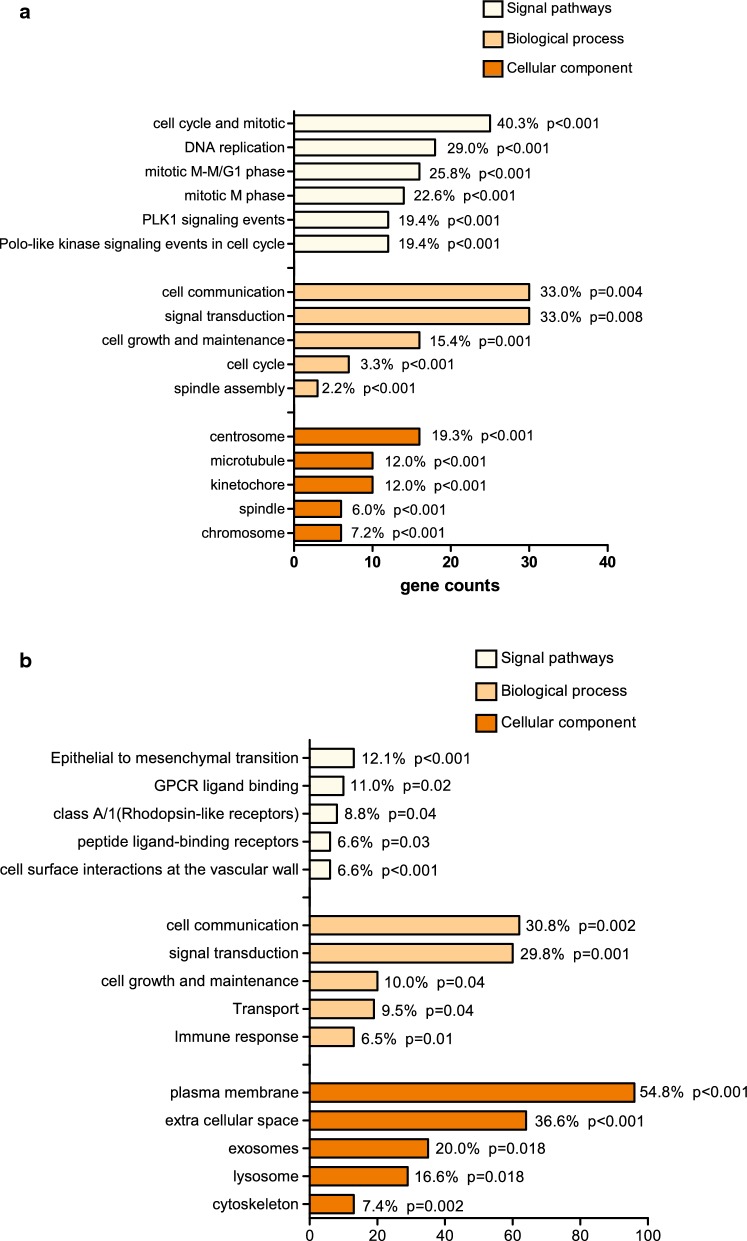
GO/KEGG analysis of 92 up-regulated and 214 down-regulated DEGs. **a** GO and KEGG reveals the main cellular components, biological processes and signaling pathways the 92 up-regulated DEGs enriched in. **b** GO and KEGG reveals the main cellular components, biological processes and signaling pathways the 214 down-regulated DEGs enriched in

The results of GO and KEGG revealed that the cellular components of 214 down-regulated DEGs were mainly enriched in plasma membrane and extracellular space, the biological processes were focused on cell communication and signal transduction, and the signaling pathways were mostly epithelial to mesenchymal transition related (Fig. 2b).

Interestingly, as for the 92 up-regulated DEGs, the cellular components were mainly focused on centrosome, spindle microtubule and chromosome region, the biological processes were enriched in spindle assembly, and the signaling pathways were mainly mitotic and DNA replication related (Fig. 2a). All molecular aspects including cell components, biological processes and signaling pathways point to the cell division process, indicating the worthy of consideration potential value of cell cycle related genes in the development of lung cancer.

### TOP2A works as the core gene in 306 DEGs PPI network

**Fig. 3:**
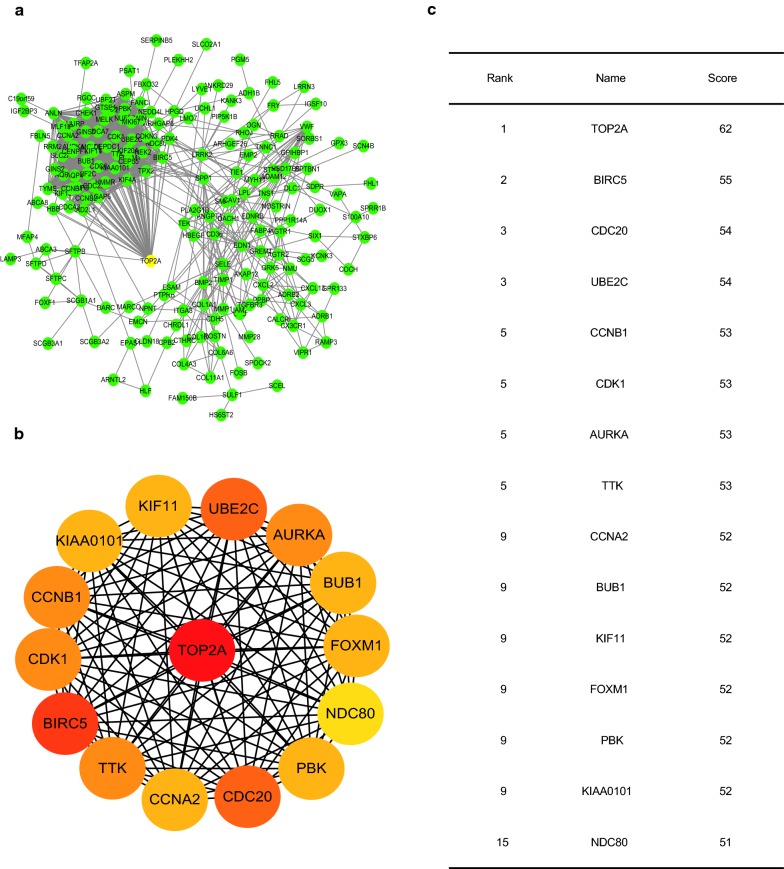
The PPI network of 306 shared DEGs. **a** The PPI network constructed for 306 DEGs. **b** Top 15 genes with high connectivity with surrounding genes (higher color represents stronger connectivity). **c** Top 15 genes with high connectivity with surrounding genes listed in descending order

To reveal the protein–protein relationship of DEGs, we constructed the PPI network of 306 shared DEGs using STRING and Cytoscape3.6.0 software (Fig. 3a). Based on the network, we identified TOP2A as the top gene with highest connectivity degree with other genes, suggesting its core position in the network (Fig. 3b, c).

**Fig. 4:**
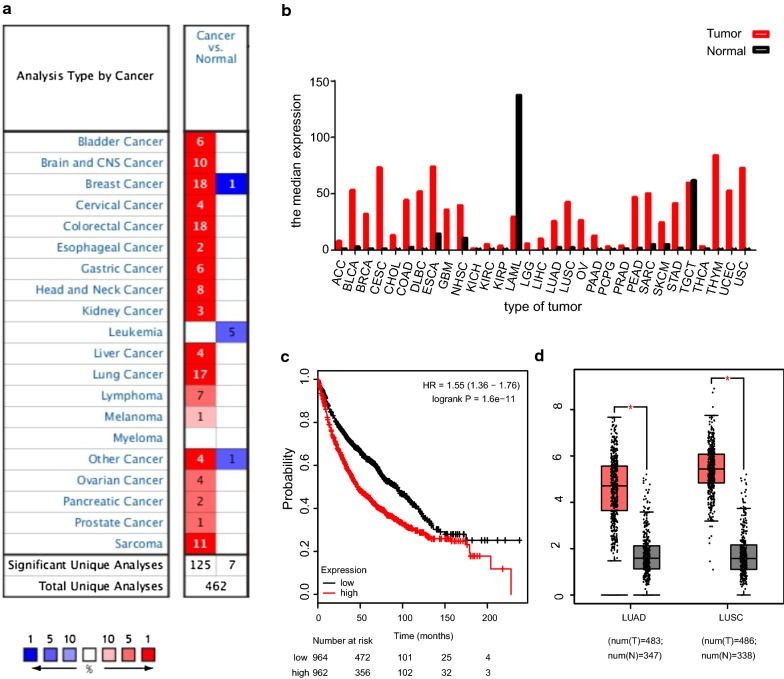
Prognosis value and expression analysis of TOP2A. **a** Expression of TOP2A in different types of human cancers. This graphic generated by Oncomine indicates the numbers of datasets with statistically significant (P < 0.01) mRNA over-expression (red) or down-expression (blue) of TOP2A (cancer vs. normal tissue). The threshold was designed with the following parameters: P-value of 1E−4, fold change of 2, and gene ranking of 10%. **b** Expression of TOP2A in different types of human cancers by GEPIA analysis. **c** Overall survival value of TOP2A by Kaplan–Meier survival analysis. **d** Aberrant gain of expression of TOP2A in NSCLC comparing to normal lung tissues, including lung adenocarcinoma (left column) squamous cancer (right column). *P < 0.05

Additionally, Kaplan–Meier plotter overall survival analysis which contains 1928 NSCLC samples revealed that TOP2A statistical significantly correlates with patients OS. Higher TOP2A expression was associated with worse NSCLC OS suggesting its probable tumor promoter function and potential survival predictor (Fig. 4c).

### Aberrant TOP2A up regulation in human NSCLC cancer

We analyzed the expression profile of TOP2A in various human tumors using Oncomine database, and the results revealed that TOP2A expression was higher in most solid tumors including lung cancer, bladder cancer, brain cancer, breast cancer, digestive tract cancers, liver cancer and many other cancers comparing to their matched normal tissues (Fig. 4a). And another analysis performed by GEPIA also showed consistent results that TOP2A was broad-spectrum up-regulated in various human tumors except for acute myeloid leukemia (Fig. 4b). Both Oncomine and GEPIA analysis suggested the aberrant gain of expression of TOP2A in NSCLC, including adenocarcinoma (483 cancer and 347 normal cases analyzed) and squamous cell carcinoma (486 cancer and 338 normal cases analyzed) comparing to normal lung tissues (Fig. 4d).

Additionally, Oncomine analysis of cancer vs normal samples revealed that TOP2A expressed statistical significantly higher in all subtypes of NSCLC, including adenocarcinoma, squamous cell carcinoma and large cell carcinoma comparing to normal tissues (Table 1).

**Table 1:** The significant changes of TOP2A expression in transcription level between different types of lung cancer and normal tissues (ONCOMINE database)

Subtype of lung cancer	P-value	Fold change	Rank (%)	Sample	References
Lung adenocarcinoma	1.65E−5	4.578	1	9	Yamagata
Squamous cell lung carcinoma	2.84E−6	4.284	1	12	Yamagata
Lung adenocarcinoma	1.13E−19	11.812	1	110	Hou
squamous cell lung carcinoma	2.34E−38	23.698	1	92	Hou
Large cell lung carcinoma	6.08E−9	24.158	1	84	Hou
Squamous cell lung carcinoma	7.26E−9	35.709	1	38	Bhattacharjee
Small cell lung carcinoma	8.63e−8	12.935	1	23	Bhattacharjee
Lung adenocarcinoma	2.17E−5	3.222	2	46	Garber
Squamous cell lung carcinoma	1.53E−6	4.478	1	19	Garber
Large cell lung carcinoma	3.82E−5	4.043	1	10	Garber

### IHC experiment validation of TOP2A gain of expression

**Fig. 5:**
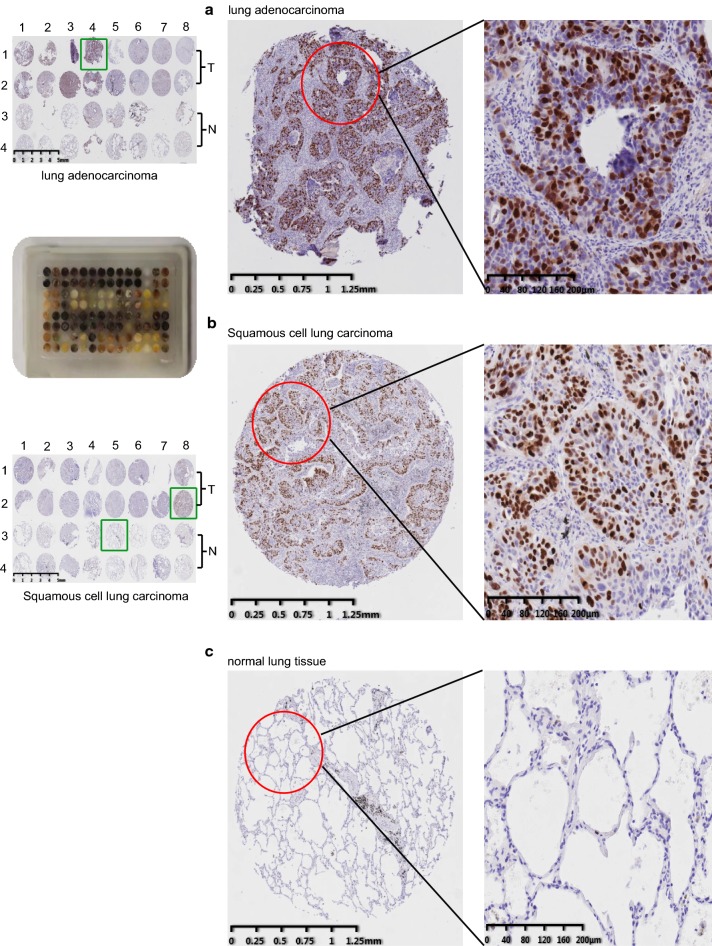
Expression level of TOP2A in NSCLC verses normal lung tissues revealed by IHC experiment. 107 Local hospitalized cancer samples were made into tissue arrays (as the left line graphics). Two spots were picked from each sample to avoid the tumor heterogeneity (in a vertical line, the first two adjacent round tissues (vertical 1, 2) is from the tumor region of one patient case, the second two adjacent tissues (vertical 3, 4) is from the matched normal FFPE block of same patient case). The number in the horizontal row meas different sample case (horizontal 1, 2, 3… is different patient cases 1, 2, 3…). The relative expression of TOP2A in qualified in **a** lung adenocarcinoma microarray, **b** squamous cell carcinoma array and **c** normal lung tissues revealed by IHC experiment

Immunohistochemistry (IHC) was carried out in 107 paired NSCLC and matched normal lung tissues (including 61 adenocarcinoma and 46 squamous cell carcinoma cases) (Fig. 5a–c). The results showed that TOP2A expression was significantly higher in cancer tissues comparing to matched normal sections. Significant TOP2A gain of expression ratio (36.4%) in NSCLC were observed by IHC staining in verse the low ratio (less than 1%) in normal tissues (P < 0.001).

Additionally, we analyzed the association between TOP2A expression and NSCLC clinicopathological parameters. Statistical analysis results showed that TOP2A positive staining ratio was higher in squamous cell carcinoma than in adenocarcinoma (P = 0.000). And TOP2A was tend to be more positively expressed in male than female patients (P = 0.001). Smoking or not also has an influence of gene expression, TOP2A was more likely to be positive in smoking patients than in non-smoking ones (P = 0.006). Meanwhile, no significance TOP2A expression differences were found regarding to patients age, tumor location, size, stages or existing evasion to bronchial tubes and lymph nodes (Table 2).

**Table 2:** The relationship between TOP2A and NSCLC clinical pathological parameters

Group	TOP2A		P value
	−	+	
Histological subtype			
Adenocarcinoma	48 (78.7%)	13 (21.3%)	P = 0.000
Squamous carcinoma	20 (43.5%)	26 (56.5%)	
Gender			
Male	35 (51.5%)	33 (48.5%)	P = 0.001
Female	33 (84.6%)	6 (15.4%)	
Age			
< 55	15 (75.0%)	5 (25.0%)	P = 0.238
≥ 55	53 (60.9%)	34 (39.1%)	
Smoke			
No	34 (79.1%)	9 (20.9%)	P = 0.006
Yes	34 (53.1%)	30 (46.9%)	
Location			
Upper lobe	31 (58.5%)	22 (41.5%)	P = 0.521
Middle lobe	7 (63.6%)	4 (36.4%)	
Lower lobe	30 (69.8%)	13 (30.2%)	
Tumor size			
< 2 cm	13 (76.5%)	4 (23.5%)	P = 0.228
≥ 2 cm	55 (61.6%)	35 (38.9%)	
Differentiation poorly			
Differentiated	13 (54.2%)	11 (45.8%)	P = 0.182
Moderately differentiated	47 (63.5%)	27 (36.5%)	
Well differentiated	8 (88.9%)	1 (11.1%)	
p-Stage			
I	22 (64.7%)	12 (35.3%)	P = 0.907
II	19 (67.9%)	9 (32.1%)	
III	11 (57.9%)	8 (42.1%)	
IV	16 (61.5%)	10 (38.5%)	
Invasion of capsule			
No	42 (68.9%)	19 (31.1%)	P = 0.190
Yes	26 (56.5%)	20 (43.5%)	
Invasion of bronchial stump			
No	63 (65.6%)	33 (34.4%)	P = 0.188
Yes	5 (45.5%)	6 (54.5%)	
LN metastasis			
−	46 (62.2%)	28 (37.8%)	P = 0.656
+	22 (66.7%)	11 (33.3%)	

### TCGA data explored independent prognostic indicator value of TOP2A in adenocarcinoma

Since the number of samples cases being used for IHC experiment was relatively low (61 cases for adenocarcinoma and 46 for squamous cell carcinoma respectively), and the number of patients with greater than 2, 3, 4 and 5 years follow-up was 70, 22, 9 and 6 respectively, the median follow-up of the 107 cases was 30 months. To avoid the limitations of relatively small number samples and short duration of follow-up, greater data of NSCLC samples were downloaded from TCGA database, including the clinical and mRNA transcription information of 574 lung adenocarcinoma and 555 lung squamous cell carcinoma for multivariate regression analysis.

**Fig. 6:**
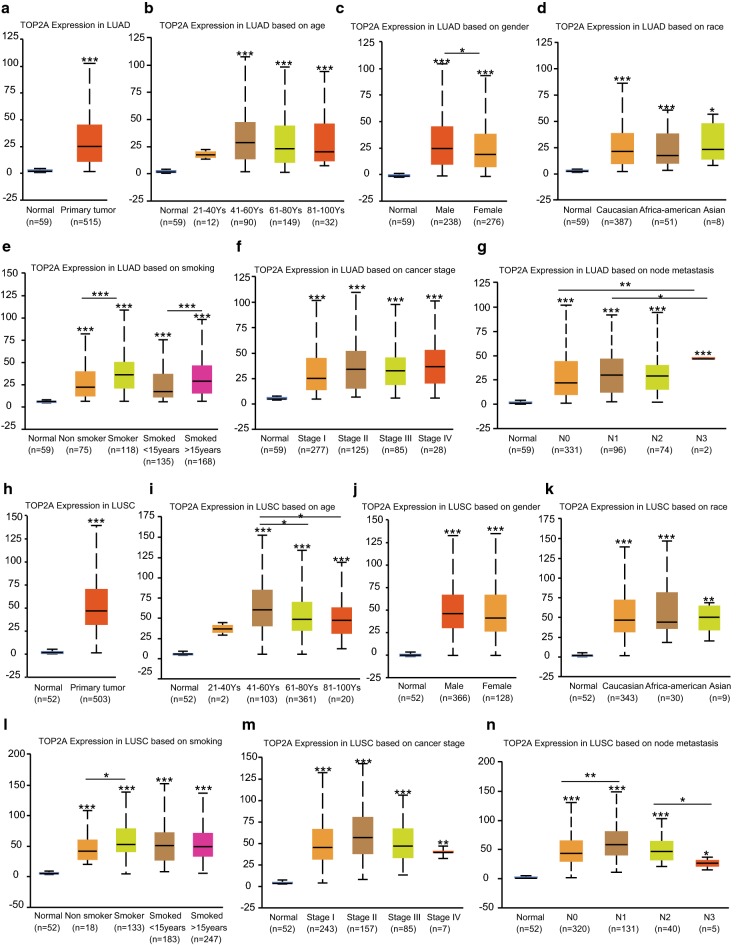
The relationship between TOP2A expression and clinical parameters. **a** Relative TOP2A expression in lung adenocarcinoma. And the association between TOP2A expression and adenocarcinoma, **b** patients age, **c** gender, **d** race, **e** smoking status, **f** tumor stage and **g** lymph node metastasis. **h** Relative TOP2A expression in lung squamous cell carcinoma. And the association between TOP2A expression and squamous cell carcinoma **i** patients age, **j** gender, **k** race, **l** smoking status, **m** tumor stage and **n** lymph node metastasis. *P < 0.05, **P < 0.01, ***P < 0.001. (The first layer asterisk which is right above the error bar representing comparison to normal group, and the above layers asterisk which were above a secondary line represent the comparison between corresponding groups that were covered by the line)

The clinical parameters based on TCGA data revealed a consistent result as our IHC experiment of local hospital patients. Besides the relatively high expression in cancers comparing to normal tissues (Fig. 6a, h), TOP2A expression was tend to be higher in male and smoker patients comparing to female and non-smokers (Fig. 6c, e, j, l). Meanwhile, no significance relationship was found between TOP2A expression and patients race (Fig. 6d, k), age (Fig. 6b, i) and cancer stage (Fig. 6f, m). Interestingly, TCGA data revealed that TOP2A expression statistical significantly associates with lymph node metastasis (N stage), that TOP2A was higher expressed in N3 comparing to N0 and N2 adenocarcinoma, and in N1, N3 comparing to N0, N2 squamous cell carcinoma. The lack of difference of TOP2A expression in different N stage of our IHC samples was considered to be of the small number of samples, especially after being sub-classed into different N stages.

Meanwhile, multivariate COX regression analysis revealed that T stage, N stage and TOP2A expression work as independent prognostic factors in lung adenocarcinoma. However, only M stage works as an independent prognostic factor in squamous cell carcinoma (Table 3).

**Table 3:** Cox proportional hazards regression on NSCLC overall survival

Variables	Lung adenocarcinoma			Lung squamous cell carcinoma		
	Hazard ratio	95% CI	P value	Hazard ratio	95% CI	P value
T						
T1 vs T2 vs T3 vs T4	1.356	1.104–1.665	0.004	1.275	0.998–1.630	0.052
N						
N_0_ vs N_1–3_	1.839	1.198–2.822	0.005	1.097	0.726–1.657	0.66
M						
M_0_ vs M_1_	1.031	0.865–1.229	0.735	1.235	1.017–1.500	0.033
TOP2A expression						
< median vs > median	0.663	0.500–0.879	0.004	1.246	0.914–1.699	0.064

### TOP2A centered signaling pathways

To preliminary understand the biological processes that TOP2A mainly participates in and the signaling pathways involving, we conducted Go and KEGG pathway analysis. Interestingly, the results showed that the top 5 biological processes TOP2A participates in were mitotic cell cycle, cell division, mitotic cell cycle process, nuclear division and chromosome segregation respectively (Table 4), and the top signaling pathways TOP2A involved were cell cycle, oocyte meiosis and progesterone-mediated oocyte maturation related (Table 5).

**Table 4:** Biological process events centered on TOP2A

Description	Counts	Background gene counts	FDR	Matching proteins in the network
Mitotic cell cycle	10	628	9.19E−12	BUB1, CCNB2, CDC20, CDK1, DLGAP5, NCAPG, PBK, TOP2A, TPX2, UBE2C
Cell division	9	483	6.67E−11	BUB1, CCNB2, CDC20, CDK1, NCAPG, TOP1, TOP2A, TPX2, UBE2C
Mitotic cell cycle process	9	564	1.76E−10	BUB1, CCNB2, CDC20, CDK1, DLGAP5, NCAPG, TOP2A, TPX2, UBE2C
Nuclear division	7	268	4.29E−09	BUB1, CDC20, DLGAP5, NCAPG, TOP2A, TPX2, UBE2C
Chromosome segregation	6	253	1.89E−07	BUB1, CDC20, DLGAP5, NCAPG, TOP1, TOP2A

**Table 5:** KEGG signaling pathways centered on TOP2A

Term description	Counts	Background gene counts	FDR	Matching proteins in the network
Cell cycle	4	123	5.57E−06	BUB1, CCNB2, CDC20, CDK1
Oocyte meiosis	4	116	5.57E−06	BUB1, CCNB2, CDC20, CDK1
Progesterone-mediated oocyte maturation	3	94	8.19E−05	BUB1, CCNB2, CDK1
p53 signaling pathway	2	68	0.0022	CCNB2, CDK1
Ubiquitin mediated proteolysis	2	134	0.0066	CDC20, UBE2C
Cellular senescence	2	156	0.0074	CCNB2, CDK1
Viral carcinogenesis	2	183	0.0086	CDC20, CDK1
HTLV-I infection	2	250	0.0137	CCNB2, CDC20

All 5 top processes and key signaling pathways pointed to the orientation of cellular mitotic regulation, indicating the vital effect TOP2A has on cell division process in vivo and the potential worthy of consideration value TOP2A working as another chemotherapy drug target, the hypothesis is based on a well known fact that most current chemotherapy drugs are developed according to their regulation on cell cycle procedures.

### Co-expression of TOP2A protein

**Fig. 7:**
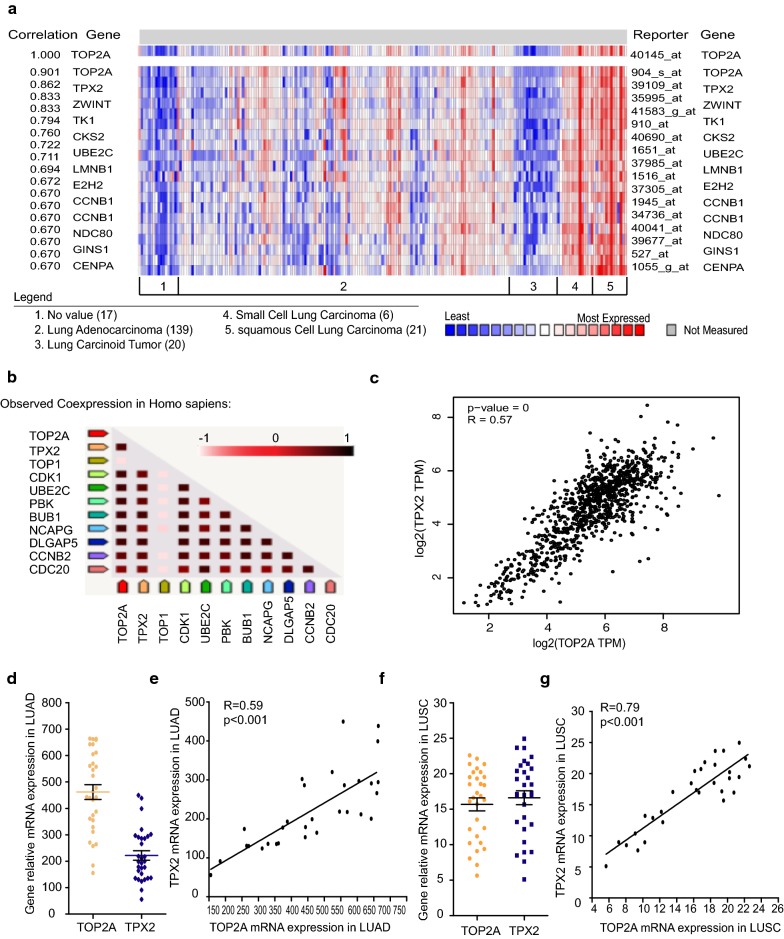
Co-expression analysis of TOP2A and TPX2. Genes that co-expressed with TOP2A revealed by **a** Oncomine database, which shows TOP2A and TPX2 association R = 0.862 and **b** PPI network, which shows TPX2 has best association with TOP2A. **c** Correlation between TOP2A and TPX2 in NSCLC revealed by GEPIA (R = 0.57). The relative expression of TOP2A and TPX2 in **d** lung adenocarcinoma and **f** squamous cell carcinoma revealed by qRT-PCR experiment. Pearson relationship between TOP2A and TPX2 in **e** lung adenocarcinoma and **g** squamous cell carcinoma

We conducted the co-expression analysis of TOP2A protein with 3 different bioinformatic tools. Firstly, we used Oncomine database which covers 8603 genes in 203 cancer samples, and we identified TPX2 as the top gene with best correlation with TOP2A, R value = 0.862 (Fig. 7a). Then, PPI network confirmed the positive correlation between TOP2A and TPX2, R value = 0.993 (Fig. 7b). Last but not least, we performed GEPIA analysis, which result revealed TPX2 working as a co-expression protein with TOP2A, P < 0.001 and R = 0.57 (Fig. 7c). All these findings suggest that TOP2A is closely related to TPX2 signaling pathways in NSCLC.

Additionally, qRT-PCR was conducted on 30 paired lung adenocarcinoma and squamous cell carcinoma samples of local hospital (different from the 107 samples used to make tissue array) to validate the relation between TOP2A and TPX2. The result revealed that both TOP2A and TPX2 expressed much higher in cancers (both LUAD and LUSC) comparing to normal lung tissues (Fig. 7d, f), and Pearson correlation analysis results showed that TOP2A expression was highly similar to TPX2, R = 0.59, 0.79 in LUAD and LUSC respectively (Fig. 7e, g).

All the bioinformatic analysis and qRT-PCR experimental results support the hypothesis that TOP2A potentially regulate NSCLC cancer development through co-work with TPX2.

## Discussion

Lung cancer is a common malignant tumor with top mortality and morbidity in both male and female cancer patients [4]. And 80–85% of lung cancer is NSCLC, which includes adenocarcinoma, squamous cell carcinoma and large cell carcinoma. Although current molecular targeted therapy and immunotherapy have been bringing promising effect for NSCLC treatment, the targets are still limited comparing to highly progressive and evolutionary cancer cells, the outcome of patients is not promising [15, 16]. The study is to identify potential prognostic indicators and new drug targets of NSCLC using bioinformatic analysis.

Bioinformatic has been a data-driven branch of science, which is commonly used for high-through data analysis and involves a large number of powerful analysis tools, software packages and database [25]. Great utilizing of these tools and software shall be an effective methodology for avoiding unnecessary repeated labour and mining useful insights buried in the high-throughput information, for instance, chips and sequencing “big-data”.

GEO database together with TCGA database, are two most commonly used databases to worldwide researchers, both databases are open-access to public and owning tremendous amount of information. In the study, we firstly chosen four cDNA expression profiles GSE18842, GSE19188, GSE33532 and GSE101929 based on the number of samples and the publication data from GEO database. The profiles contains a total of 249 NSCLC and 119 normal samples, and GEO2R tool was then used to analyze the DEGs between cancer and normal tissues, discovering that 306 DEGs were shared in all four profiles, including 214 down-regulated and 92 up-regulated genes. GO and KEGG analysis revealed that most of the 92 up-regulated DEGs were focused on cell cycle and cell division related signaling.

To better understand the internal relationship of 306 genes, PPI network was constructed. And top 15 genes with strongest connection with other genes were identified, including TOP2A, BIRC5, CDC20, UBE2C, CCNB1, CDK1, AURKA, TTK, CCNA2, BUB1, KIF11, FOXM1, PBK, KIAA0101 and NDC80. Out of the 15 genes, TOP2A possess best connection with surroundings.

TOP2A, which is short for Topoisomerase II Alpha, locates at 17q21.2 and encodes an enzyme that controls and alters the topological states of DNA during transcription. This enzyme has been known to be involved in processes such as chromosome condensation, chromatid separation, and the relief of torsional stress that occurs during DNA transcription and replication. A most well known disease associated with TOP2A is female breast cancer, it is usually deleted or amplified simultaneously with ERBB2, thus the two genes are commonly co-tested in breast cancer patients for further proper use of anticancer agent herceptin [44–46]. And, TOP2A was reported to be targeted by tumor suppressor like miR-144-3p in glioblastoma, thus resulting in cancer cell apoptosis [47]. As in lung cancer, Pabla et al. [48] reported that TOP2A could be a potential new indicator in PD-L1 negative NSCLC, however, deeper analysis is still needed for mechanism explanation. In the study, we analyzed TOP2A function in NSCLC development using bioinformatic tools.

Firstly, Kaplan–Meier plotter overall survival analysis was used to reveal the correlation between TOP2A and NSCLC OS, and the results showed that TOP2A statistical significantly correlates with patients OS, higher TOP2A expression was associated with worse OS. And Multivariate Cox regression analysis supported TOP2A expression works as an independent prognostic indicator in lung adenocarcinoma, suggesting its probable tumor promoter and potential survival indicator function in further clinical use.

Then, to validate the aberrant gain of expression of TOP2A in NSCLC, GEPIA was firstly performed, and the results showed that TOP2A was up-regulated in cancers comparing to normal tissues. Our IHC experiment which was conducted on 107 cases of local hospitalized NSCLC patients surgery samples also confirmed the results, significant TOP2A gain of expression ratio (36.4%) in NSCLC was observed by IHC staining in verse the low ratio (less than 1%) in normal tissues. Meanwhile, clinical significance analysis showed that TOP2A expression was associated with cancer subtype, patients gender and smoking. TCGA data also supported the association between TOP2A expression and clinical parameters including patients gender and smoking status.

Additionally, TOP2A involving signaling pathways revealed that its main function in NSCLC is also cell cycle regulation related, consistent with the previous GO/KEGG analysis of up-regulated DEGs in NSCLC. And three different analyzing software including Oncomine database, PPI network and GEPIA software all predicted the positive correlation between TOP2A and TPX2, and qRT-PCR experiment conducted on 30 paired local hospital adenocarcinoma and squamous cell carcinoma samples validated the association between two genes, indicating TPX2 is a probable co-working partner of TOP2A.

TPX2, locates at 20q11.21, is one of the main spindle assembly factors that play a key role in inducing microtubule assembly and growth during M phase of mitosis [49–51]. Previous studies reported that TPX2 mRNA expression during cell cycle progression is high in G2/M phase, decreases dramatically upon G1 phase entry, increases upon entry into S phase, and peaks again at the next G2/M phase [52–56]. The drop in TPX2 is consistent with the drastic reorganization in structure and dynamics of the mitotic spindle [57]. Due to its important role in microtubule assembly and mitosis, TPX2 has been found to be over expressed in various human cancers, for instance clear renal cell carcinoma [58], esophageal carcinoma [59], hepatocellular carcinoma (HCC) [52, 60], gastric cancer [61], bladder carcinoma [62] and so on. TPX2 expression has been shown to be positively correlated with poor prognosis, metastasis, and recurrence [49, 63].

However, above results aren’t yet enough to put TOP2A or TPX2 as a drug target in NSCLC, to distinguish gene aberrations that can cause the disease and may serve as drug targets with those only closely linked to the disease and consequently are associated with the disease development, comprehensive and longitudinal experiments, as well as clinical trials are needed to be performed.

## Conclusion

In summary, using bioinformatic analysis, we analyzed 306 DEGs between NSCLC and normal lung tissues, and TOP2A was identified as the core gene in the network. IHC experiment validated the aberrant gain of expression of TOP2A in cancer comparing to normal tissues. OS analysis revealed the association between TOP2A expression and worse prognosis. Additionally, TOP2A could be effected on NSCLC cell cycle progression through co-working with TPX2. Large-scale and comprehensive studies are needed to confirm the findings before promoting the clinical utility of TOP2A as a prognosis indicator and drug target.

## Data Availability

All data generated or analyzed during this study are included in this published article.

## Abbreviations

NSCLC: non-small cell lung cancer
SCLC: small cell lung cancer
GEO: Gene Expression Omnibus
DEGs: differently expressed genes
PPI: protein–protein interaction
OS: overall survival rate
STRING: Search Tool for the Retrieval of interacting Genes
MCODE: molecular complex detection
GO: gene ontology
KEGG: Kyoto Encyclopedia of Gene and Genome
IHC: immunohistochemistry
TCGA: The Cancer Genome Atlas
EGFR: epidermal growth factor receptor
ALK: anaplastic lymphoma kinase
TOP2A: Topoisomerase II Alpha
